# Supplementary material for: Flipping antimicrobial peptides in the exit tunnel of the bacterial ribosome
Source: Nat Commun. 2026 Jun 3;17:4914. doi: 10.1038/s41467-026-74007-x (PMC13233979; doi:10.1038/s41467-026-74007-x)
Supplement: Supplementary file 1 — Supplementary information [file 41467_2026_74007_MOESM1_ESM.pdf]

Flipping antimicrobial peptides in the exit tunnel of the bacterial ribosome

Weiping Huang , Max J. Berger, Haaris A. Safdari, Dorota Klepacki, Helge Paternoga, Chetana Baliga, Daniel N. Wilson, Nora Vazquez-Laslop, Alexander S. Mankin

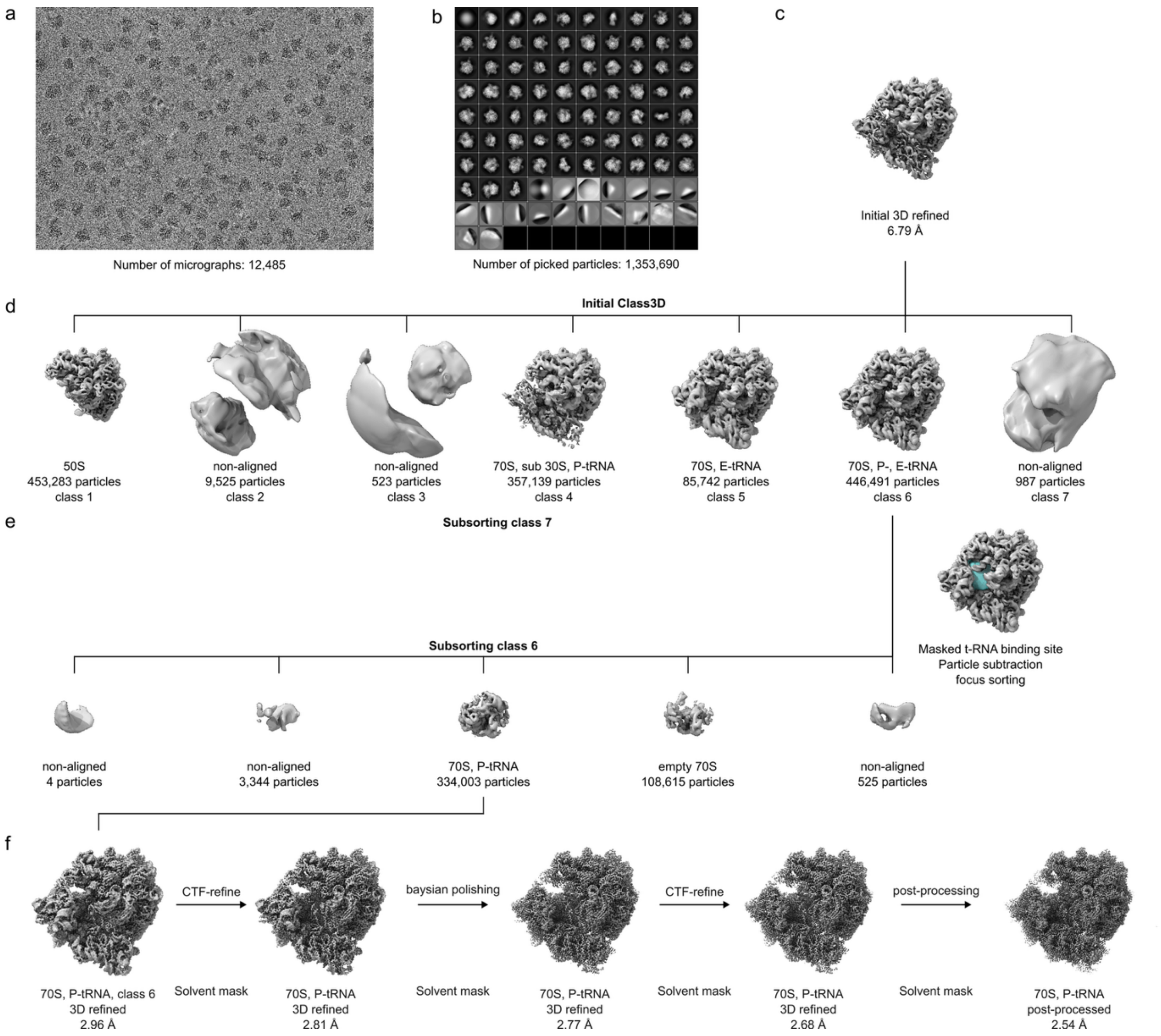

Supplementary Figure 1. *In silico* sorting scheme of the *E. coli* Dal2-70S complex. a, representative micrograph for the *E. coli* Dal2-70S complex. **b**, From 12,485 micrographs, 1,353,690 ribosome-like particles were selected after 2D-classification. **c**, Particles were 3D refined at 4x decimated pixel size. **d**, Initial 3D classification for 200 iterations led to seven subclasses. **e,** The resulting P-tRNA containing class 6 was subjected to 200 iterations of focused 3D classification with a mask around the tRNA binding sites. **f**, The 70S complex containing P-tRNA density was 3D refined at undecimated pixel size and subjected to CTF refinement (4th order aberrations, beam-tilt, anisotropic magnification and per-particle defocus value estimation), Bayesian polishing and again CTF refined, subsequent post-processing resulted in a final average resolution for the masked reconstruction of 2.5 Å (at $FSC_{0.143}$).

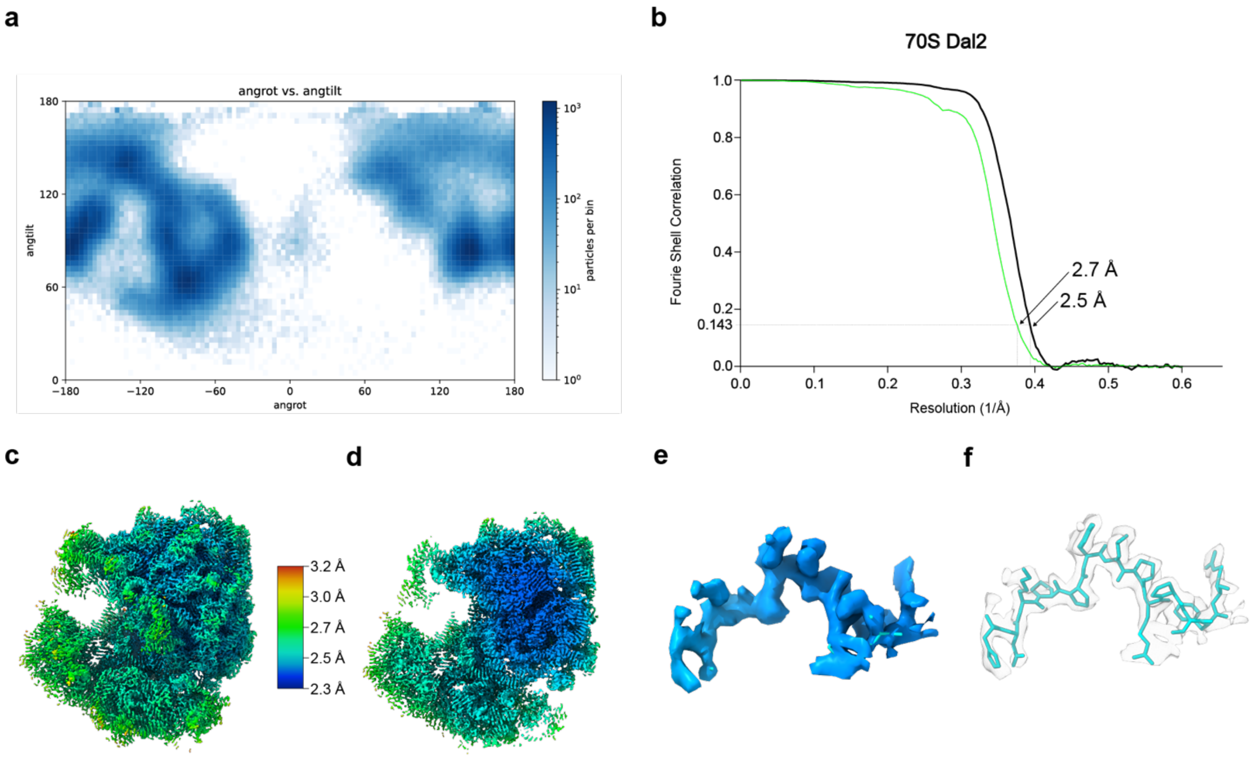

Supplementary Figure 2. Angular distribution, FSC curve and local resolution of the Dal2-70S complex. (**a**) Angular distribution of the particles that comprise the Dal2-70S complex. (**b**) Fourier shell correlation (FSC) curve (masked, black and unmasked, green) of the Dal2-70S complex. (**c-d**) Cryo-EM map colored according to the local resolution of the Dal2-70S map (**c**) with transverse section (**d**). (**e-f**) Cryo-EM density of Dal2, (**e**) colored according to the local resolution, and (**f**) shown as mesh with molecular model (aqua).

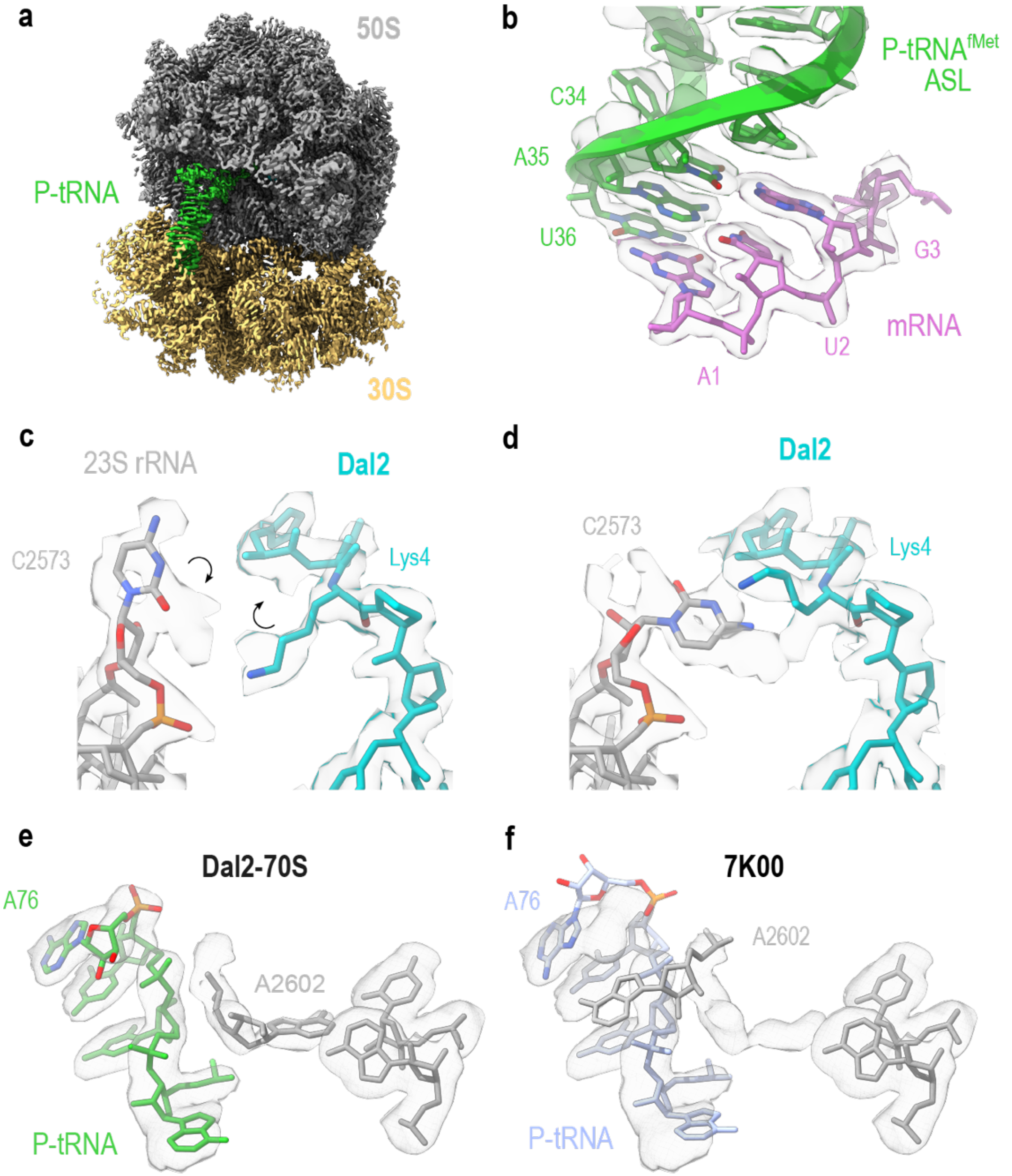

Supplementary Figure 3. Binding of Type I PrAMP Dal2 affects the placement of the P-site tRNA and conformation of rRNA in the PTC. a, Cryo-EM map of the Dal2-70S complex with P-site tRNA (green), 30S (yellow) and 50S (grey). **b**, Cryo-EM density (transparent) and molecular model for the anticodon stem loop (ASL) of the P-site bound fMet-tRNA (green) interacting with the AUG start codon of the mRNA (pink). **c-f,** Density (transparent) and molecular model for the alternative conformations of (**c,d**) 23S rRNA nucleotide C2573 (grey) and the sidechain of Lys4 of Dal2 (cyan), as well as (**e,f**) 23S rRNA nucleotide A2602 (grey) of the 23S rRNA relative to P-site tRNA (green) in the (**e**) Dal2-70S complex, compared to (**f**) the conformation of 23S rRNA nucleotide A2602 (grey) relative to P-site tRNA (blue) in the canonical 70S ribosome (PDB ID 7K00)[1].

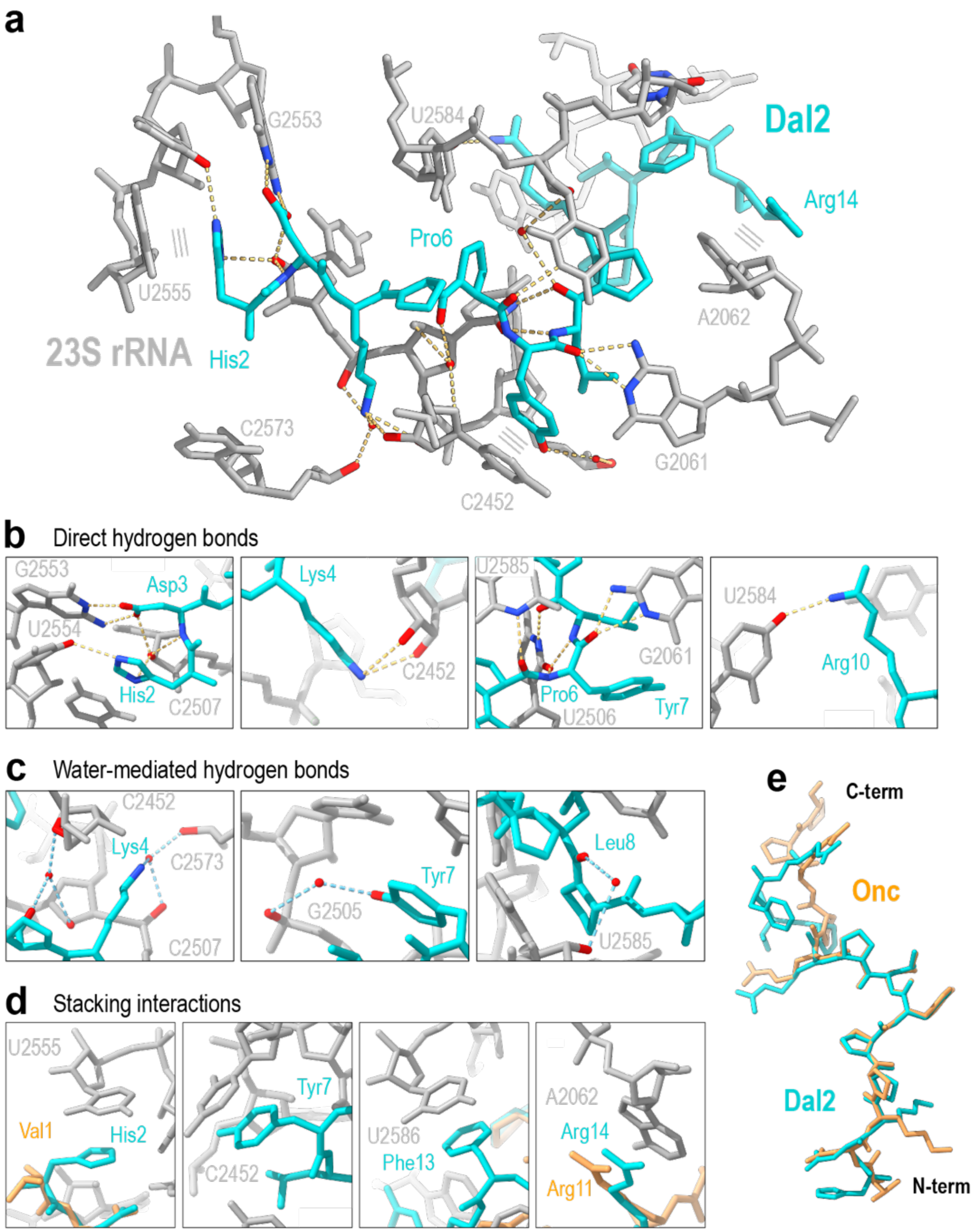

Supplementary Figure 4. Interactions of Dal2 with the ribosome and comparison with Onc112. (**a**) Overview of the interactions of Dal2 (light blue) with the *E. coli* 23S rRNA residues (grey) in the Dal2-70S complex. Dashed lines indicate potential hydrogen bond interactions. (**b-d**) Dal2-ribosomes interactions include (**b**) Direct hydrogen interactions (orange dashed lines), (**c**) water-mediated hydrogen bonds (cyan dashed lines with waters indicated by red spheres), and (**d**) stacking interactions between Dal2 (cyan) or oncocin (Onc, orange, PDB ID 5HCR)[2] with 23S rRNA nucleotides. (**e**) Comparison of Dal2 and Onc binding position within the NPET. Structural alignment based on the 23S rRNA.

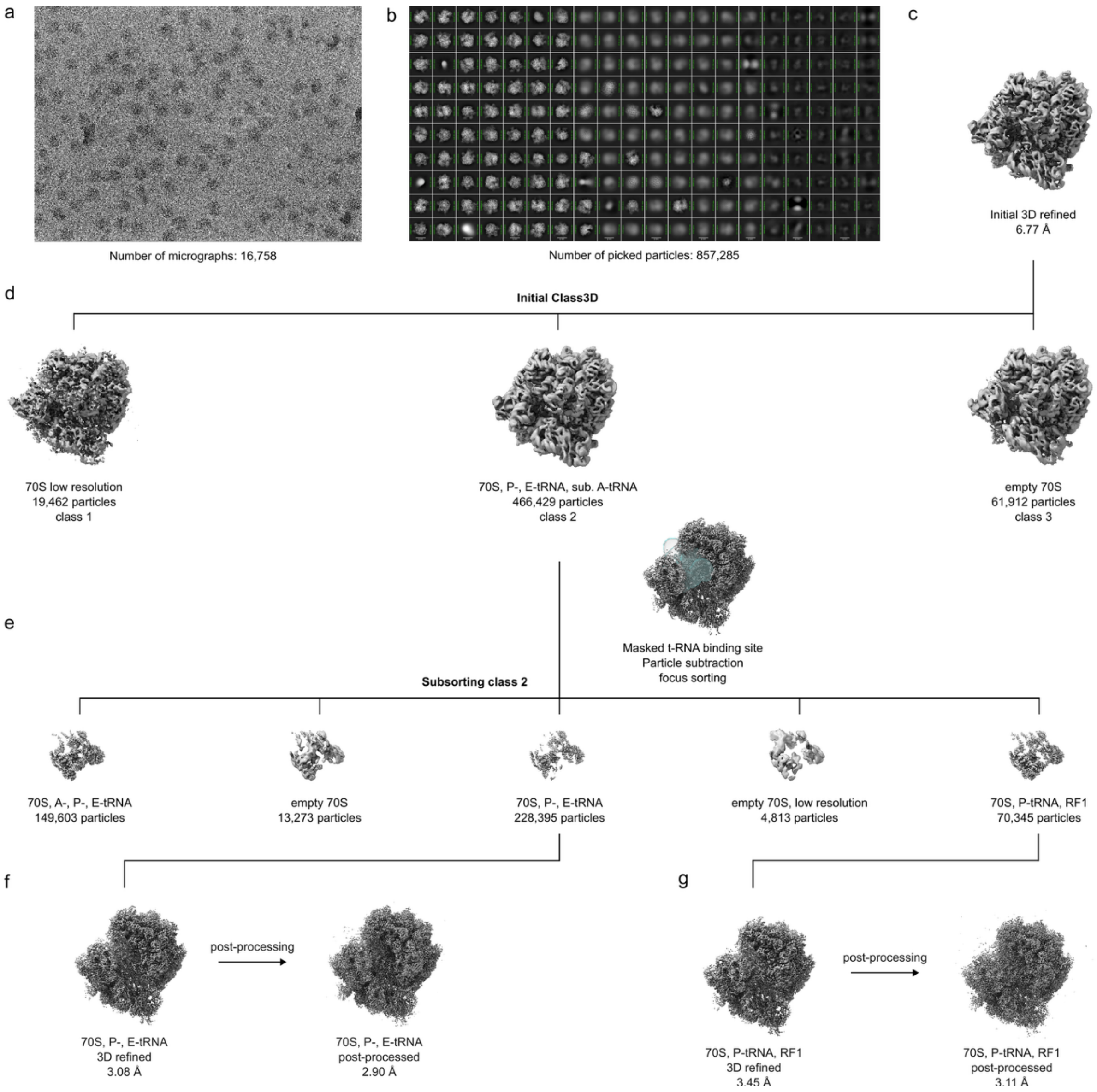

Supplementary Figure 5. *In silico* sorting scheme of the *E. coli* DalDro complex. a, representative micrograph for the *E. coli* DalDro-70S complex. **b**, From 16,758 micrographs, 857,285 ribosome-like particles were selected after 2D-classification. **c**, Particles were 3D refined at 4x decimated pixel size. **d**, Initial 3D classification for 200 iterations led to three subclasses. **e,** The resulting class2 containing P-, E-tRNA and sub stoichiometric A-tRNA was subjected to 200 iterations of focused 3D classification with a mask around the tRNA binding sites. **f**, The 70S complex containing P- and E-site tRNA density was 3D refined at undecimated pixel size and subsequently post-processed, resulting in a final average resolution for the masked reconstruction of 2.9 Å (at $FSC_{0.143}$). **g,** The complex containing P-tRNA and RF1 was refined at undecimated pixel size. Subsequent post-processing led to a final resolution for the masked reconstruction of 3.1 Å (at $FSC_{0.143}$).

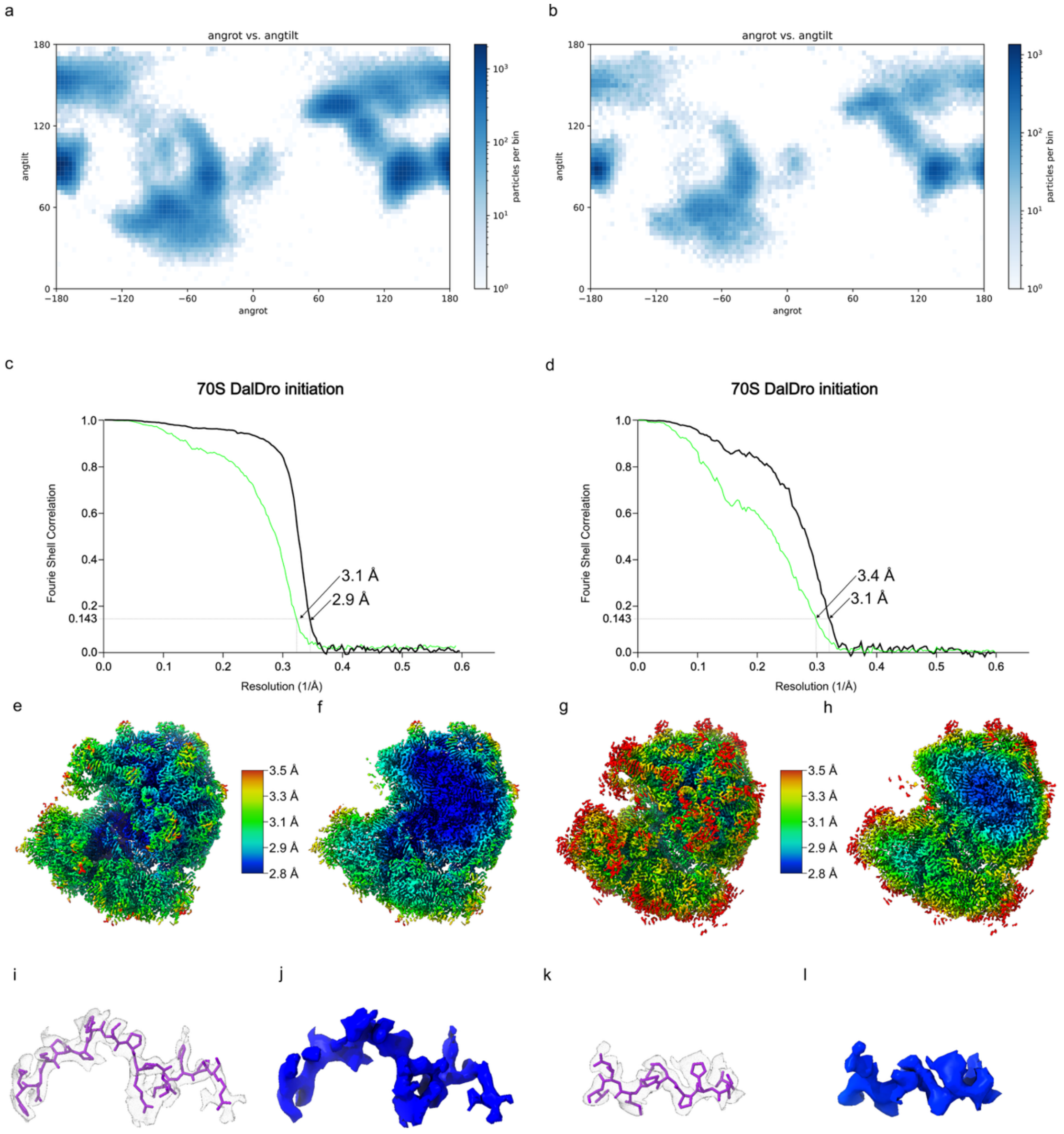

Supplementary Fig. 6. Angular distribution, FSC and local resolution of DalDro complex. a-b, Angular distribution of the particles that comprise the (**a**) DalDro-70S initiation state and (**b**) DalDro-70S termination state. **c-d**, Fourier shell correlation (FSC) curves of the (**c**) 70S refined cryo-EM map of the DalDro-70S initiation state and (**d**) the DalDro-70S termination state. **e-h**, Cryo-EM density map colored according to local resolution for (**e**) DalDro-70S initiation state with (**f**) transverse section, and (**g**) the DalDro-70S termination state with (**h**) transverse section. **i-l**, Cryo-EM density for DalDro from the (**i-j**) DalDro-70S initiation state and (**k,l**) DalDro-70S termination state, shown as (**i,k**) mesh with molecular model or (**j,l**) colored according to local resolution.

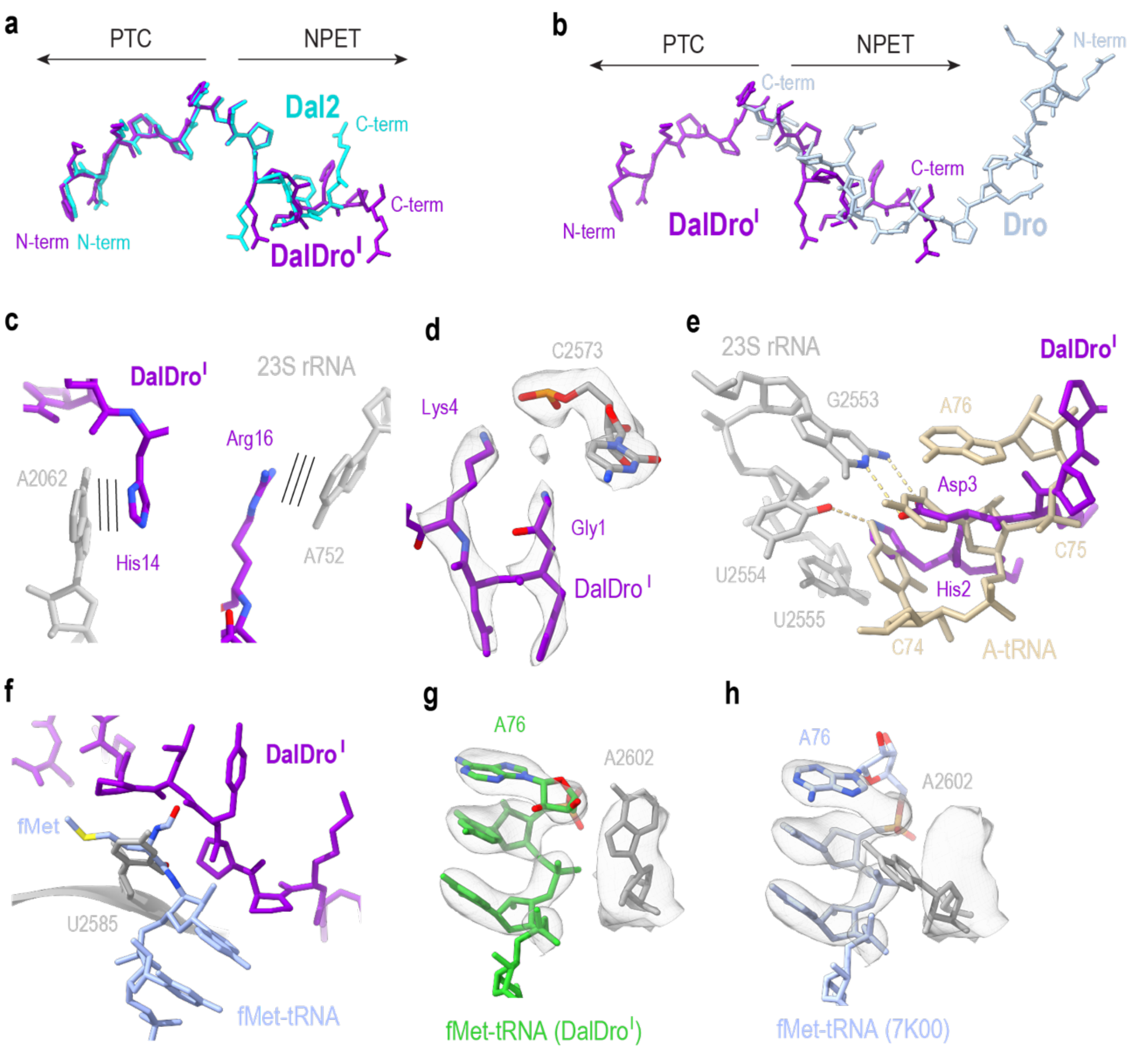

Supplementary Figure 7. Interactions of DalDro in the Type I (DalDro^{I}) orientation with the ribosome. a-b, Comparison of binding mode of DalDro^{I} (magenta) with (**a**) Dal2 (cyan) and (**b**) Drosocin (Dro, light blue) (PDB ID 8AKN)[3]. Alignments were based on 23S rRNA. **c,** Stacking interactions of DalDro^{I} His14 with A2062, and of Arg16 with A752 of the 23S rRNA. **d,** Position of Gly1 of DalDro^{I} (magenta) located between Lys4 of DalDro^{I} and nucleotide C2573 (grey) of the 23S rRNA. **e,** N-terminus of DalDro^{I} is stabilized by interactions between His2 and Asp3 of DalDro^{I} (magenta) with G2553 and U2554 (grey) of the 23S rRNA, mimicking the interactions of C74 and C75 of the CCA end of the A-tRNA (tan). **f,** U2585 (grey) adopts a position in the presence of DalDro^{I} (magenta) that is incompatible with the canonical position of the fMet moiety of the initiator tRNA (pale blue). **g,** Cryo-EM density for the CCA-end of the P-site tRNA (green) and A2602 (grey) in the DalDro^{I}-70S complex. **h,** as in (**g**), but with the model for the canonical CCA-end of an fMet-tRNA and position of A2602 from a 70S ribosome (PDB ID 7K00) [1].

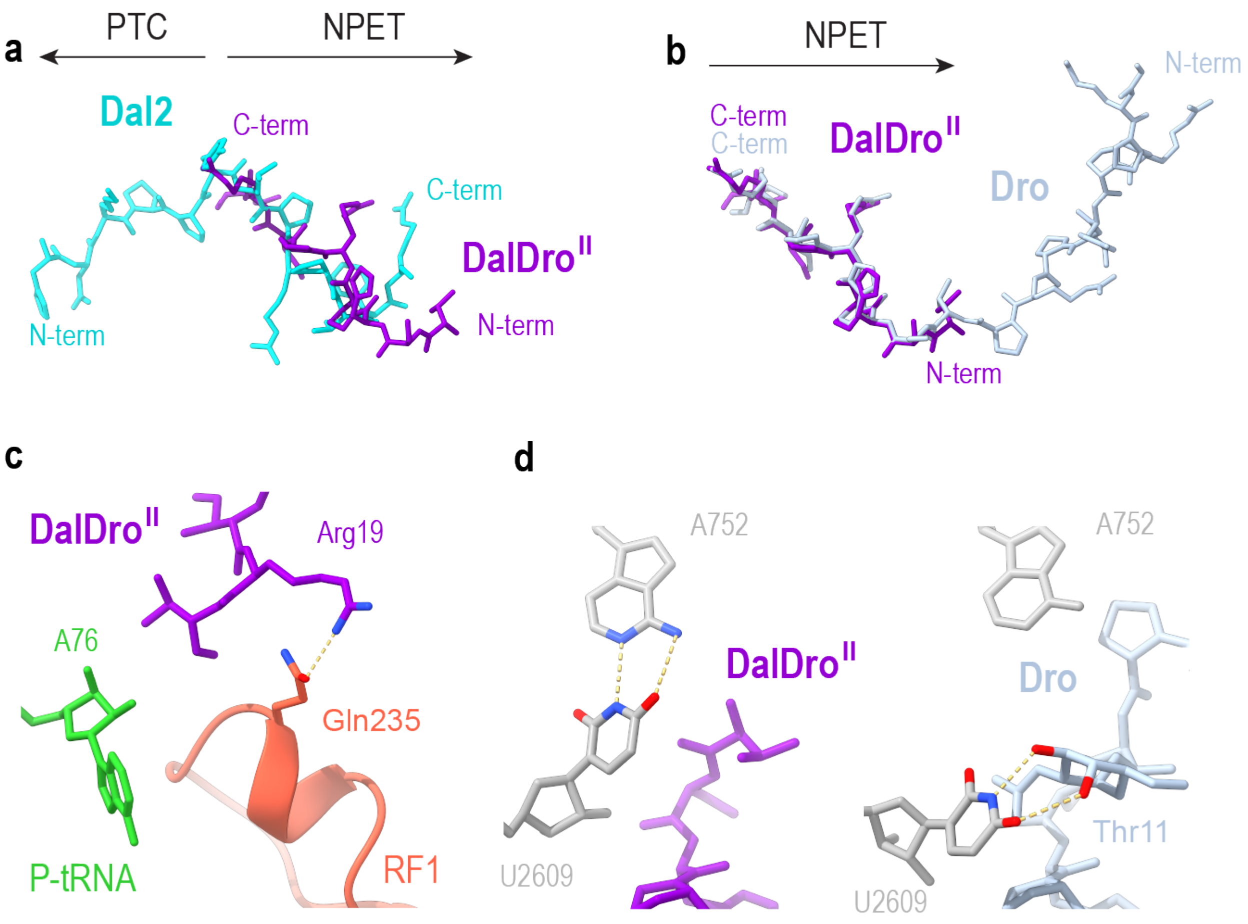

Supplementary Figure 8. Interactions of DalDro in the Type II (DalDro^{II}) orientation with the ribosome. a-b, Comparison of binding mode of DalDro^{II} (magenta) with (**a**) Dal2 (cyan) and (**b**) Dro (light blue) (PDB ID 8AKN)[3]. Alignments were based on 23S rRNA. **c,** Potential hydrogen bond (dashed yellow line) formed between Arg19 of DalDro^{II} (magenta) and Gln235 of RF1 (red) relative to A76 of the P-site tRNA (green). **d,** (left) In the presence of the non-glycosylated peptide DalDro^{II} (magenta) base pairing can form between 23S rRNA nucleotides A752 and U2609 (grey), whereas (right) the A752 and U2609 base-pair is broken due to a movement of A752 that is induced by the binding of the glycosylated form of Dro (pale blue) (PDB ID 8AKN), as reported [3].

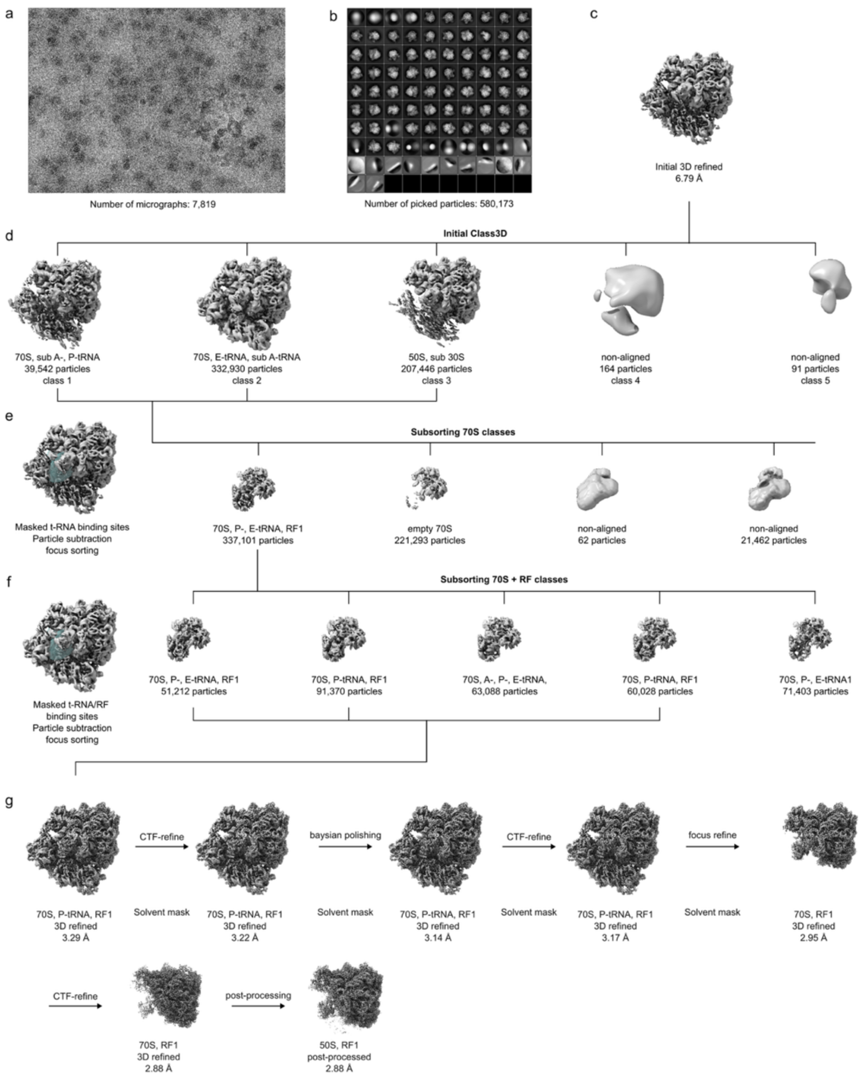

Supplementary Fig. 9. *In silico* sorting scheme of the *E. coli* DalDroS complex. a, representative micrograph for the *E. coli* DalDroS-70S complex. **b**, From 7,819 micrographs, 580,173 ribosome-like particles were selected after 2D-classification. **c**, Particles were 3D refined at 4x decimated pixel size. **d**, Initial 3D classification for 200 iterations led to five subclasses. **e-f,** The classes containing 70S were subjected (**e**) to 200 iterations of focused 3D classification with (**e**) a mask around the tRNA binding sites. The class containing RF1 was further subsorted (**f**) with a mask around tRNA and RF1 binding sites. **g**, All classes containing 70S complex and RF1 density were combined and 3D refined at undecimated pixel size, subjected to CTF refinement (4th order aberrations, beam-tilt, anisotropic magnification and per-particle defocus value estimation), Bayesian polishing and again CTF refined. 70S particles were subtracted with a mask around the 50S subunit and subsequent 3D refinement resulted in a final average resolution for the masked reconstruction of 2.9 Å (at $FSC_{0.143}$).

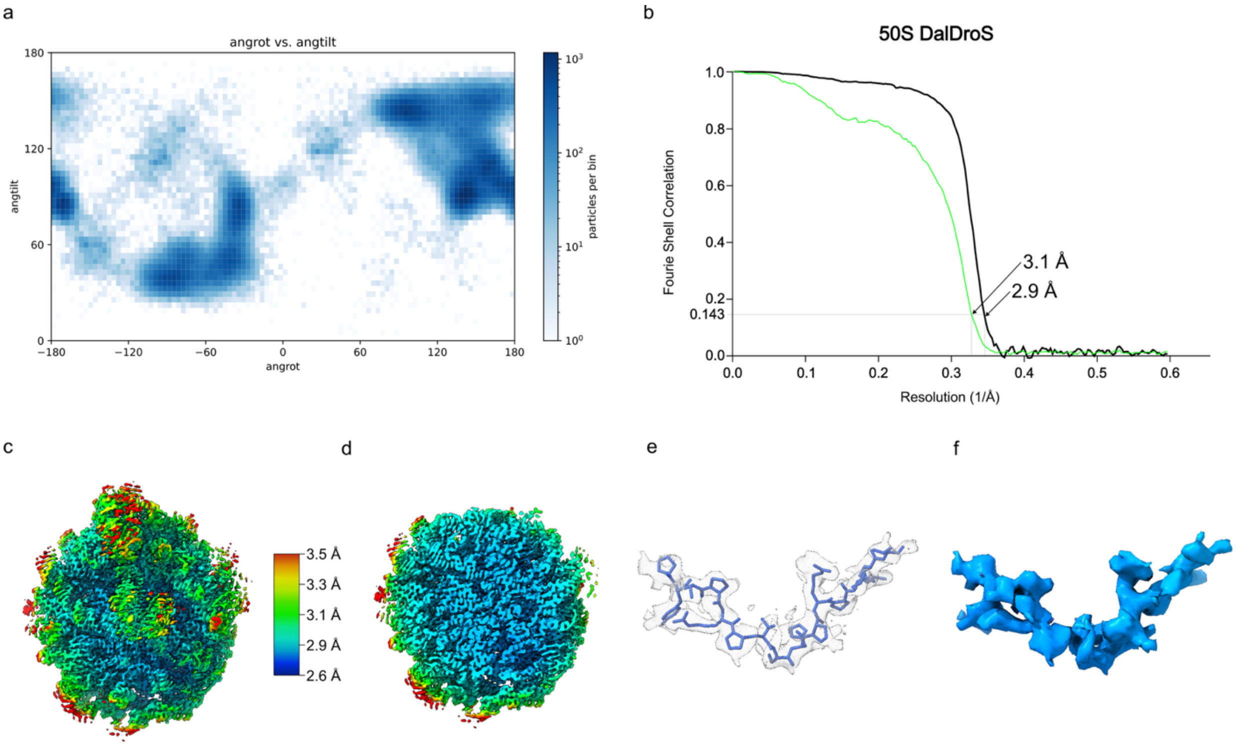

Supplementary Fig. 10. Angular distribution, FSC curve and local resolution of the DalDroS 70S cryo-EM map. (**a**) Angular distribution of the particles that comprise the DalDro bound 50S focus refined ribosome map. (**b**) Fourier shell correlation (FSC) curve (masked, black and unmasked, green) of the 50S DalDroS bound complex. (**c-d**) Cryo-EM map colored according to the local resolution of the 50S map (**c**) with transverse section (**d**). (**e-f**) Cryo-EM density of modelled DalDroS residues, shown as mesh including the molecular model (**e**) and colored according to the local resolution (**f**).

Supplementary Table 1. Fly proteins that specify Drosocin-like PrAMPs

Species	Peptide ID	Non-redundant isoforms of PrAMPs	Non-redundant PrAMP-specifying proteins from the genus *Drosophila*	Repeats / isoforms	Source
Drosophila innubila	Din1	YERPPYLPRPTPRPYARV	>XP_034478517.1 apidaecins type 22-like isoform X1 [*Drosophila innubila*]<br>MKFTITLLLAAICVMAVS**GHERPPYLPRPTFRPYARV**VREADP**GYERPPYLPRPTFRPYARV**VREADPGSEISVSRVVREADPA**YERPPYLPRPTPRPYARV**VREANPA**YERPPYLPRPTPRPYARV**VREADP**GHERPPYLPRPTFRPFRV**VREASPENVPMIEDVYQY	5	NCBI
	Din2	GHERPPYLPRPTFRPFRV			
	Din3	GYERPPYLPRPTFRPYARV			
Drosophila albomicans	Dal1	GHDKPPYLPRPTFRPISRV	>XP_034107673.1 apidaecins type 73-like [*Drosophila albomicans*]<br>MKFAITLLLLSICALAVVS**GHERPPYLPRPTFRPYSRA**VREADP**GHDKPPYLPRPTFRPVSRV**VREADP**GHDKPPYLPRPTFRPVARV**VREADP**GHDKPPYLPRPTFRPVARV**VREADP**GHDKPPYLPRPTFRPISRV**VREADP**GHDKPPYLPRPTFRPVARV**VREADP**GHDKPPYLPRPTFRPVARV**VREADP**GHDKPPYLPRPTFRPVARV**VREADP**GHDKPPYLPRPTFRPVSRV**VREADP**GHDKPPYLPRPTFRPVARV**VREADPGHDKPLHLRVVHEENPGQVARVVREADPGHERPPYLPRPTFRPYS	10	NCBI
	Dal2	GHDKPPYLPRPTFRPVSRV			
	Dal3	GHDKPPYLPRPTFRPVARV			
	Dal4	GHERPPYLPRPTFRPYSRA			
Drosophila grimshawi	Dgr1	HERPPYLPRPTFRPYARL	>EDW00936.1_*Drosophila grimshawi*<br>MNFSIVILLLSLFAMALA**HERPPYLPRPTFRPYARL**VREAIPVADPVNLEVEQF	1	NCBI
	Dgr2	YERPPYRPRPTFRPYSRL	>gnl|dgri|scaffold_15245 _dbxref=GB:CH916367<br>MNFSIIFLLLSLFAVALA**YERPPYRPRPTFRPYSRL**VREAIPVDNTLSVETQQL	1	Fly database
	Dgr3	YERPPYRPRPTFRPYSRL	>gnl|dgri|scaffold_15245 _dbxref=GB:CH916367<br>MNFSIIFLLLSLFAVALA**YERPPYRPRPTFRPYSRL**VREAIPVDNTLNVETQQL	1	
Drosophila hydei	Dhy1	GYERPPYLPRPTFRPIHRV	>XP_030078547.1 uncharacterized protein LOC115482760 [*Drosophila hydei*]<br>MKFTIIFLLLSMCAMALA**GYERPPYLPRPTFRPVRI**VREA**GYERPPYLPRPTFRPVHRF**VREA**GYERPPYLPRPTFRPIHRV**VREALPAAEQLWADAPQF	3	NCBI
	Dhy2	GYERPPYLPRPTFRPVRI			
	Dhy3	GYERPPYLPRPTFRPVHRF			
Drosophila arizonae	Dar1	GYERPPYRPRPTFRPVHRI	>XP_017867847.1 PREDICTED: uncharacterized protein LOC108616858 [*Drosophila arizonae*]<br>MKFTIILLLVSMCAMAMA**GYERPPYRPRPTFRPVHRI**ARAA**GYERPPYLPRPTFRPVRI**ARAA**DYERPPYLPRPTFRPINRY**VREAPPAELLQEDALQF	3	NCBI
	Dar2	GYERPPYLPRPTFRPVRI			
	Dar3	DYERPPYLPRPTFRPINRY			
Drosophila navojoa	Dna1	GYERPPYRPRPTFRPVHRI	>XP_017961159.1 uncharacterized protein LOC108654385 [*Drosophila navojoa*]<br>MKFTIILLLVSMCAMAMA**GYERPPYRPRPTFRPVHRI**ARA**AGYERPPYLPRPTFRPIRI**ARAA**DYERPPYLPRPTFRPINRY**VREAPPAELLQEDTLQF	3	NCBI
	Dna2	GYERPPYLPRPTFRPIRI			
	Dna3	DYERPPYLPRPTFRPINRY			
Drosophila willistoni	Dwi1	GKPRPYSPRPTSHPRPIRV	>XP_002062774.1 drosocin [*Drosophila willistoni*]<br>MKFSIIFAVFLAICCALAVATP**GKPRPYSPRPTSHPRPIRV**RRDINLPLEQGQQIN	1	NCBI
Drosophila obscura	Dob1	GKPRPSNPRPTSHPRPIRV	>XP_022225789.2 drosocin [*Drosophila obscura*]<br>MKFGLVFLLLACIWCLSAATP**GKPRPSNPRPTSHPRPIRV**RREAMAIVDRLELIQPAVAARSPPILPA	1	NCBI
Drosophila subobscura	Dsu1	AKPRPNNPRPTSHPRPIRV	>XP_034653632.1 drosocin [*Drosophila subobscura*]<br>MKFSLVFLLLACIWALATASP**AKPRPNNPRPTSHPRPIRV**RREALAIADHLELIQPAVAAKSPSILAA	1	NCBI
Drosophila kikkawai	Dki1	GKPKPYSPRPTSTPRPIRV	>XP_017024457.1 drosocin [*Drosophila kikkawai*]<br>MKFTIVFLLLACVFALAMATP**GKPKPYSPRPTSTPRPIRV**RREALAIDDHLGLVQAAAAIRPPPILPA	1	NCBI
Drosophila serrata	Dse1	GKPKPYSPRPTSHPRPIRV	>KAH8375251.1 hypothetical protein KR200_003426 [*Drosophila serrata*]<br>MKFTIVFLLLACVFAMAMATP**GKPKPYSPRPTSHPRPIRV**RREALAIDDQLGLVQAAAAIKPPPILPL	1	NCBI
Drosophila jambulina	Dja1	GQPKPYSPRPTSHPRPIRV	>KAH8290752.1 hypothetical protein KR054_005605 [*Drosophila jambulina*]<br>MKFTIVFLLLACVFALAMATP**GQPKPYSPRPTSHPRPIRV**RRDQLGLVKPAPTRLPPVQTF	1	NCBI
Drosophila elegans	Del1	GKPRPISPRPTSHPRPIRV	>XP_017133075.2 attacin-B isoform X2 [*Drosophila elegans*]<br>MKFTIIFLLLACVLAMAVATP**GKPRPISPRPTSHPRPIRV**RREVLGGSLASNPAGGADARVDLTKGIGSPDHNLVGQVFAAGNTQGGPVTSGGTLAYNNAGHGASLTRTHTPGVKDVFQQEAHANLFNNGRHNLDAKVFASQNKLANGFEFQRNGAGLDYSHINGHGASLTHSNFPGIGQQLGAEGRANLWSSADRATRLDLTGSASKWTSGPFANQRTNFGAGLGLSHHFG	1	NCBI
Drosophila biarmipes	Dbi1	GKPKPYSPRPTSHPRPIRV	>XP_016946682.1 drosocin [*Drosophila biarmipes*]<br>MKFTIVFLLLACVFAMALATP**GKPKPYSPRPTSHPRPIRV**RREAVTVEDHLAQAAIRPPPILPA	1	NCBI
Drosophila melanogaster	Dro	GKPRPYSPRPTSHPRPIRV	>NP_001246324.1 drosocin, isoform B [*Drosophila melanogaster*]<br>MKFTIVFLLLACVFAMGVATP**GKPRPYSPRPTSHPRPIRV**RREALAIEDHLTQAAIRPPPILPA	1	NCBI
Drosophila mojavensis	Dmo1	GYERPPYRPRPTFRSVHRI	>gnl|dmoj|scaffold_6496 dbxref=GB:CH933808<br>MKFTIILLLVSMCAMAMA**GYERPPYRPRPTFRSVHRI**ARAA**GYERPPYLPRPTFRPVRI**ARAA**DYERPPYLPRPTFRPINRY**VREAPPVELLQEDALQF	3	Fly databse
	Dmo2	GYERPPYLPRPTFRPVRI			
	Dmo3	DYERPPYLPRPTFRPINRY			
	Dmo4	GHDKPPYLPRPTYRPVGRI	>gnl|dmoj|scaffold_6496 dbxref=GB:CH933808<br>MNFTIILLLLSICALALA**GHDKPPYLPRPTYRPVGRI**ARAAPPAAENN	1	
Drosophila virilis	Dvi1	GHERPPYLPRPTFRPIGRV	>gnl|dvir|scaffold_12875_dbxref=GB:CH940648<br>MKLTIILLLLSICAMALA**GHERPPYLPRPTFRPIGRV**VREALPAAEPANVEAQQL	1	Fly databse
			>gnl|dvir|scaffold_12875_dbxref=GB:CH940648<br>MKFTIILLMLSICAMALA**GHERPPYLPRPTFRPIGRV**VREALPAADPANVDAQQL	1	
Drosophila pseudoobscura	Dps1	GKPRPSNPRPTSHPRPIRV	>XP_001360616.1_*Drosophila_pseudoobscura*<br>MKFSLVFLLLACIWALAAATP**GKPRPSNPRPTSHPRPIRV**RREALHIADRLGLIQPAVAAMSPPIRPA	1	NCBI
Drosophila persimilis	Dpe1	GKPRPSNPRPTSHPRPIRV	>XP_002016189.1 drosocin [*Drosophila persimilis*]<br>MKFSLVFMLLACIWALAAATP**GKPRPSNPRPTSHPRPIRV**RREALHIADRLGLIQPAVAAMSPPIRPA	1	NCBI
Drosophila ananassae	Dan1	GKPRPYSPRPTSHPRPIRV	>XP_001960757.1_drosocin_*Drosophila_ananassae*<br>MKFTIVFLLLACIFAVALATP**GKPRPYSPRPTSHPRPIRV**RREALAIEDHLGLVQPAAVVRPPPILPV	1	NCBI
Drosophila yakuba	Dya1	GKPRPYSPRPTSHPRPIRV	>XP_002091345.1_drosocin_*Drosophila_yakuba*<br>MKFTIVFLLLACVFAMAVATP**GKPRPYSPRPTSHPRPIRV**RREALATEDHLAAAAFRPPPILPA	1	NCBI
Drosophila erecta	Der1	GKPRPYSPRPTSHPRPIRV	>XP_001975597.1 _*Drosophila_erecta*<br>MKFTIVFLLLACVFAMAVATP**GKPRPYSPRPTSHPRPIRV**RREALAIEDHLAPAAFRPPPILPV	1	NCBI
Drosophila simulans	Dsi1	GKPRPYSPRPTSHPRPIRV	>XP_002081537.1_*Drosophila_simulans*<br>MKFTIVFLLLACVFAMAVATP**GKPRPYSPRPTSHPRPIRV**RREARSIEDHLAQAAIRPPPILPA	1	NCBI
Drosophila neotestacea	Dne1	GHERPPYLPRPTFRPYARV	>*Drosophila_neotestacea* MKCTFTLLIAIFAMAVS**GHERPPYLPRPTFRPYARV**VREAD	1	ref.5
	Dne2	GHERPPYLPRPTFRPYARV	>*Drosophila_neotestacea*_JAWNMD010000270.1<br>MKCTFTLLLIAIFAMAVS**GHERPPYLPRPTFRPYARV**VREVD	1	NCBI
	Dne3	GHERPPYLPRPTFRPYARV	>*Drosophila_neotestacea*_JAWNMD010000270.1<br>MKCIISLLLVAIFAMAVS**GHERPPYLPRPTFRPYARV**VREAD	1	NCBI

Supplementary Table 2. Non-redundant Dro-like PrAMPs identified in fly genomes

Species	PrAMP	Sequence
Drosophila melanogaster	Dro	GKPRPYSPRPTSHPRPIRV
Drosophila innubila	Din1	YERPPYLPRPTPRPYARV
Drosophila innubila	Din2	GHERPPYLPRPTFRPFRV
Drosophila innubila	Din3	GYERPPYLPRPTFRPYARV
Drosophila albomicans	Dal1	GHDKPPYLPRPTFRPISRV
Drosophila albomicans	Dal2	GHDKPPYLPRPTFRPVSRV
Drosophila albomicans	Dal3	GHDKPPYLPRPTFRPVARV
Drosophila albomicans	Dal4	GHERPPYLPRPTFRPYSRA
Drosophila grimshawi	Dgr1	HERPPYLPRPTFRPYARL
Drosophila grimshawi	Dgr2	YERPPYRPRPTFRPYSRL
Drosophila hydei	Dhy1	GYERPPYLPRPTFRPIHRV
Drosophila hydei	Dhy2	GYERPPYLPRPTFRPVHRF
Drosophila arizonae	Dar	GYERPPYLPRPTFRPVRI
Drosophila navojoa	Dna1	GYERPPYRPRPTFRPVHRI
Drosophila navojoa	Dna2	GYERPPYLPRPTFRPIRI
Drosophila subobscura	Dsu	AKPRPNNPRPTSHPRPIRV
Drosophila kikkawai	Dki	GKPKPYSPRPTSTPRPIRV
Drosophila serrata	Dse	GKPKPYSPRPTSHPRPIRV
Drosophila jambulina	Dja	GQPKPYSPRPTSHPRPIRV
Drosophila elegans	Del	GKPRPISPRPTSHPRPIRV
Drosophila mojavensis	Dmo1	GYERPPYRPRPTFRSVHRI
Drosophila mojavensis	Dmo2	DYERPPYLPRPTFRPINRY
Drosophila mojavensis	Dmo3	GHDKPPYLPRPTYRPVGRI
Drosophila virilis	Dvi	GHERPPYLPRPTFRPIGRV
Drosophila persimilis	Dpe	GKPRPSNPRPTSHPRPIRV
Drosophila neotestacea	Dne	GHERPPYLPRPTFRPYARV

Supplementary Table 3. Chemically synthesized peptides tested in this study

PrAMP	Sequence	Purity
Dro	GKPRPYSPRPTSHPRPIRV	87.6
Din1	YERPPYLPRPTPRPYARV	98.8
Din2	GHERPPYLPRPTFRPFRV	94.8
Dal1	GHDKPPYLPRPTFRPISRV	98.0
Dal2	GHDKPPYLPRPTFRPVSRV	90.0
Dal3	GHDKPPYLPRPTFRPVARV	97.5
Dhy1	GYERPPYLPRPTFRPIHRV	96.1
Dna1	GYERPPYRPRPTFRPVHRI	95.8
Dse	GKPKPYSPRPTSHPRPIRV	70.9
Del	GKPRPISPRPTSHPRPIRV	85.5
DroDal	GKPRPYSPRPTFRPVSRV	96.5
DalDro	GHDKPPYLPRPTSHPRPIRV	92.7
DalDroΔH	GDKPPYLPRPTSHPRPIRV	88.0
DalDroΔD	GHKPPYLPRPTSHPRPIRV	88.9
DalDroΔHD	GKPPYLPRPTSHPRPIRV	96.9
DalDroR	GHDKPRPYLPRPTSHPRPIRV	83.8
DalDroS	GHDKPPYSPRPTSHPRPIRV	89.6

Supplementary Table 4. Cryo-EM data collection, modelling and refinement statistics

Model	Dal2	DalDro initiation	DalDro termination	DalDroS
**EMDB ID**	EMD-55845	EMD-55844	EMD-55847	EMD-55849
**PDB ID**	9TEX	9TEW	9TEY	9TEZ
**Data collection and processing**				
Magnification (×)	105 000	105 000	105 000	105 000
Electron fluence (e^{-}/Å^2)	40	40	40	40
Defocus range (µm)	-1.98 – 21.4	-1.98 – 21.4	-1.98 – 21.4	-1.98 – 21.4
Pixel size (Å)	0.832	0.83	0.83	0.83
Initial particles	1 353 690	857 285	857 285	580 173
Final particles	334 003	228 395	70 345	232 610
Average resolution (Å) (FSC threshold 0.143)	2.5	2.9	3.1	2.9
**Model composition**				
Atoms	139 713	139 683	141 573	90 140
Protein residues	5570	5573	5843	3440
RNA bases	4473	4471	4461	2948
**Refinement**				
Map CC around atoms	0.96	0.93	0.89	0.92
Map CC whole unit cell	0.95	0.92	0.89	0.92
Map sharpening B factor (Å^2)	-69.87	-94.27	-85.56	-54.48
**R.M.S. deviations**				
Bond lengths (Å)	0.011	0.011	0.011	0.012
Bond angles (°)	1.405	1.449	1.468	1.585
**Validation**				
MolProbity score	0.95	0.88	1.24	1.16
Clash score	1.89	1.22	2.00	1.57
Poor rotamers (%)	0.35	0.57	0.59	0.57
**Ramachandran statistics**				
Favoured (%)	98.19	97.80	96.07	96.23
Outlier (%)	0.09	0.02	0.14	0.03

Supplementary Information References

1 Watson, Z. L. *et al.* Structure of the bacterial ribosome at 2 Å resolution. *eLife* 9 (2020).

2 Gagnon, M. G. *et al.* Structures of proline-rich peptides bound to the ribosome reveal a common mechanism of protein synthesis inhibition. *Nucleic Acids Res* 44, 2439-2450 (2016).

3 Koller, T. O. *et al.* Structural basis for translation inhibition by the glycosylated drosocin peptide. *Nat Chem Biol* 19, 1072-1081 (2023).

4 Hanson, M. A., Hamilton, P. T. & Perlman, S. J. Immune genes and divergent antimicrobial peptides in flies of the subgenus Drosophila. *BMC Evol Biol* 16, 228 (2016).
